# Diversity, Composition and Resilience of the Root Microbiome of Tomato Plants in a Hydroponic Rockwool System

**DOI:** 10.1111/1758-2229.70380

**Published:** 2026-06-21

**Authors:** Phil Thomas, Brian Sindel, Gal Winter

**Affiliations:** ^1^ School of Science and Technology University of New England Armidale New South Wales Australia; ^2^ School of Environmental and Rural Science University of New England Armidale New South Wales Australia

**Keywords:** community ecology, hydroponic, microbiome, rhizosphere

## Abstract

In hydroponic horticulture, where soil is replaced by a sterile artificial substrate, attention to microorganisms is primarily focused on the suppression of plant pathogens and the application of potentially beneficial organisms. This study examined the ecology of the root microbiome of tomato plants which were grown to maturity in a hydroponic rockwool system and then treated with a *Trichoderma*‐based biocontrol product. The bacterial community was species‐rich but was dominated by a small number of Alphaproteobacteria, Gammaproteobacteria and Bacteroidia species. The fungal community was less diverse and consisted almost exclusively of Ascomycota and Rozellomycota species. Biocontrol treatment did not have a significant effect on bacterial diversity, but the microbiome composition changed distinctly over the period of sampling in both treated and untreated plants. These results support the view that mineral substrates in a hydroponic system can support a complex and resilient root microbiome. Understanding the microorganisms that thrive in this unique environment may help identify effective biological treatments, and enable the development of rockwool‐specific practices for monitoring, protecting and promoting plant health.

## Graphical abstract

The root microbiome of tomatoes in hydroponic rockwool was species‐rich but dominated by a few abundant organisms. Treatment with a *Trichoderma*‐based biocontrol product did not perturb the bacterial microbiome. Composition and diversity of the bacterial community changed over the sampling period in both treated and control samples.

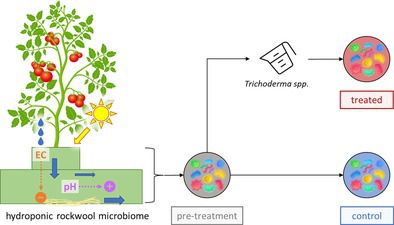

## 1 Introduction

The ‘rhizobiome’, the microbial community that inhabits the root zone of plants, has a significant influence on plant health (Berendsen et al. 2012; Trivedi et al. 2020). A beneficial rhizobiome can promote plant growth by enhancing nutrient availability and releasing phytohormones, and can also protect plants by suppressing root pathogens and stimulating the plant immune system (Backer et al. 2018; Wei et al. 2015). Plants are genetically primed to shape the rhizobiome by recruiting beneficial microorganisms from the vast diversity present in soil (Berendsen et al. 2012; Zhalnina et al. 2018). Microbial traits that are enriched in the rhizosphere include attachment mechanisms, biofilm formation, chemotaxis and elicitation of root exudates (Levy et al. 2018). A growing body of research in horticultural systems has identified the potential of the rhizobiome to control root disease (Thomas et al. 2024), stimulate resistance to foliar herbivores (Blundell et al. 2020; Pineda et al. 2017), influence the nutritional quality of fruit (Escobar Rodríguez et al. 2021) and provide early warning of plant disease (Gu et al. 2022; Lee et al. 2021).

In hydroponic horticulture, growers aim to protect plants from soil‐borne pathogens by replacing soil with a sterile substrate. Despite this aseptic beginning, soilless systems are colonised by microbes, especially bacteria, soon after planting (Berkelmann et al. 1993; Koohakan et al. 2004; Thomas et al. 2023; Vallance et al. 2012). Furthermore, the moist, temperate conditions in a glasshouse favour many species which cause diseases that affect crop yield (Paulitz 1997; Vallance et al. 2011), and plants lack access to the wide variety of potential beneficial microbes found in soil (Paulitz 1997; Raaijmakers et al. 2009). Comparisons between soil and soilless culture (Anzalone et al. 2022) and between different types of soilless substrate (Grunert et al. 2016; Guevara et al. 2025) have established that the plant growth medium has a strong effect on the bacterial and fungal composition of the root microbiome. Artificial substrates, such as the chemically inert, fibrous mineral matrix known as rockwool, are initially aseptic (Berkelmann et al. 1993) and contain no residual organic matter (Vallance et al. 2011). Organic hydroponic substrates, such as coconut fibre, provide some nutrients and carbon sources and consequently promote distinctly different microbial community diversity (Anzalone et al. 2022; Grunert et al. 2016). In contrast, the highly varied environment of a typical soil, with diverse residual organic matter and other nutrients, supports a diverse range of microbes ready to colonise roots (Bais et al. 2006; Zhalnina et al. 2018). However, the rhizobiome in hydroponic systems has received much less attention from both growers and researchers than that of soil‐grown plants. Furthermore, many studies in this area focus on specific pathogens or beneficial organisms of interest, rather than the ecosystem as a whole (e.g., de Freitas and Taylor 2023; Khalil et al. 2009; Postma et al. 2009).

The root zone of a hydroponic system provides a fundamentally different microbial environment to soil; therefore, a different microbial ecology is naturally expected (Grunert et al. 2016). Nutrients and organic matter are introduced to the system when irrigation is applied and plant roots begin to colonise the substrate (Vallance et al. 2011). Growers have extensive control over the root zone environment in a hydroponic system, for example by selection of rootstock and scion varieties, modulation of glasshouse daytime and night‐time temperatures and humidity or regulation of the composition, pH and frequency of fertigation solution applied (Alsanius and Wohanka 2019). Irrigation schedules are designed to avoid conditions that favour pathogens which promote root disease. Furthermore, growers often apply products containing microorganisms reputed to stimulate plant growth (biostimulants) and/or reduce the incidence of pathogens (biocontrols). *Trichoderma* species are commonly employed for this purpose due to their ability to suppress pathogenic microorganisms and promote plant growth (Contreras‐Cornejo et al. 2016). *Trichoderma* inoculation has been observed to significantly reduce bacterial diversity and change community composition of the rhizosphere in soil (Lee Díaz et al. 2024), but the potential impact on the rhizobiome in hydroponic systems has not been investigated. In contrast to other crop management processes, the outcomes of both deliberate and inadvertent influence on the microbiome are difficult to assess, largely because this ecosystem is both complex and difficult to observe. Understanding the ecology of the hydroponic rhizobiome—the patterns of interactions between plants, microbes and their shared environment—can help to identify cultivation practices that optimise the beneficial and protective potential of the microorganisms that live around plant roots (Thomas et al. 2023).

The aims of this study were to characterise the ecology of the root microbiome of tomato plants in a commercially relevant rockwool hydroponic system, and to observe the effect of a *Trichoderma*‐based biocontrol product on the diversity and composition of the microbiome. The extraction process and analysis targeted mainly bacteria, which are much more abundant than fungi in the rockwool environment (Koohakan et al. 2004). Sampling methods were designed to extract microorganisms from the rockwool matrix and root surfaces while minimising plant material present during DNA extraction.

## 2 Materials and Methods

### 2.1 Experimental Hydroponic System

Grafted seedlings ( *Solanum lycopersicum* cultivar ‘Endeavour’ [Rijik Zwaan] grafted on rootstock ‘DRO141’ [De Ruiter]) were first grown in a nursery on rockwool propagation cubes (Plantop, Grodan) before being planted on rockwool slabs (GT Master, Grodan) in a temperature‐controlled glasshouse with drip irrigation which was designed to replicate a commercial rockwool hydroponic system (University of New England, NSW, Australia). A total of 24 plants were planted in the same glasshouse compartment on two parallel hydroponic channels. Each channel supported four rockwool slabs with three plants per slab. An open (drain‐to‐waste) drip irrigation system was used to supply municipal water with two‐part fertiliser added by proportional injectors to provide complete plant nutrition. Plants were irrigated only during daylight, and the frequency and duration of irrigation shots were adjusted according to the time of day and light intensity, following the same principles as a commercial glasshouse. As plants matured, the electrical conductivity (EC) and pH of the irrigation solution were adjusted to the levels used in a commercial glasshouse (target pH 5.5–6.5, target EC 2.8–3.0 mS cm^−1^). Glasshouse temperature was initially set to 25°C daytime/18°C night, then adjusted to 23°C daytime/16°C night when the first trusses were mature. A typical schedule of commercial plant maintenance was performed (including pollinating, hanging, twisting, pruning and picking).

At 68 days after planting (DAP), when the first trusses were mature, four randomly selected slabs (two per channel) were treated with a commercial biocontrol agent containing multiple strains of *Trichoderma* (PlantMate, Agrimm, New Zealand) according to the supplier instructions. Briefly, 180 mg of product was mixed in 10 L of tap water and applied by pouring onto the propagation cubes of plants in the four treatment slabs. The remaining four slabs were maintained as a control group under the same growing conditions. The manufacturer of the biocontrol product recommends reapplication every 10–14 days.

### 2.2 Hydroponic System Sampling and DNA Extraction

**FIGURE 1:**
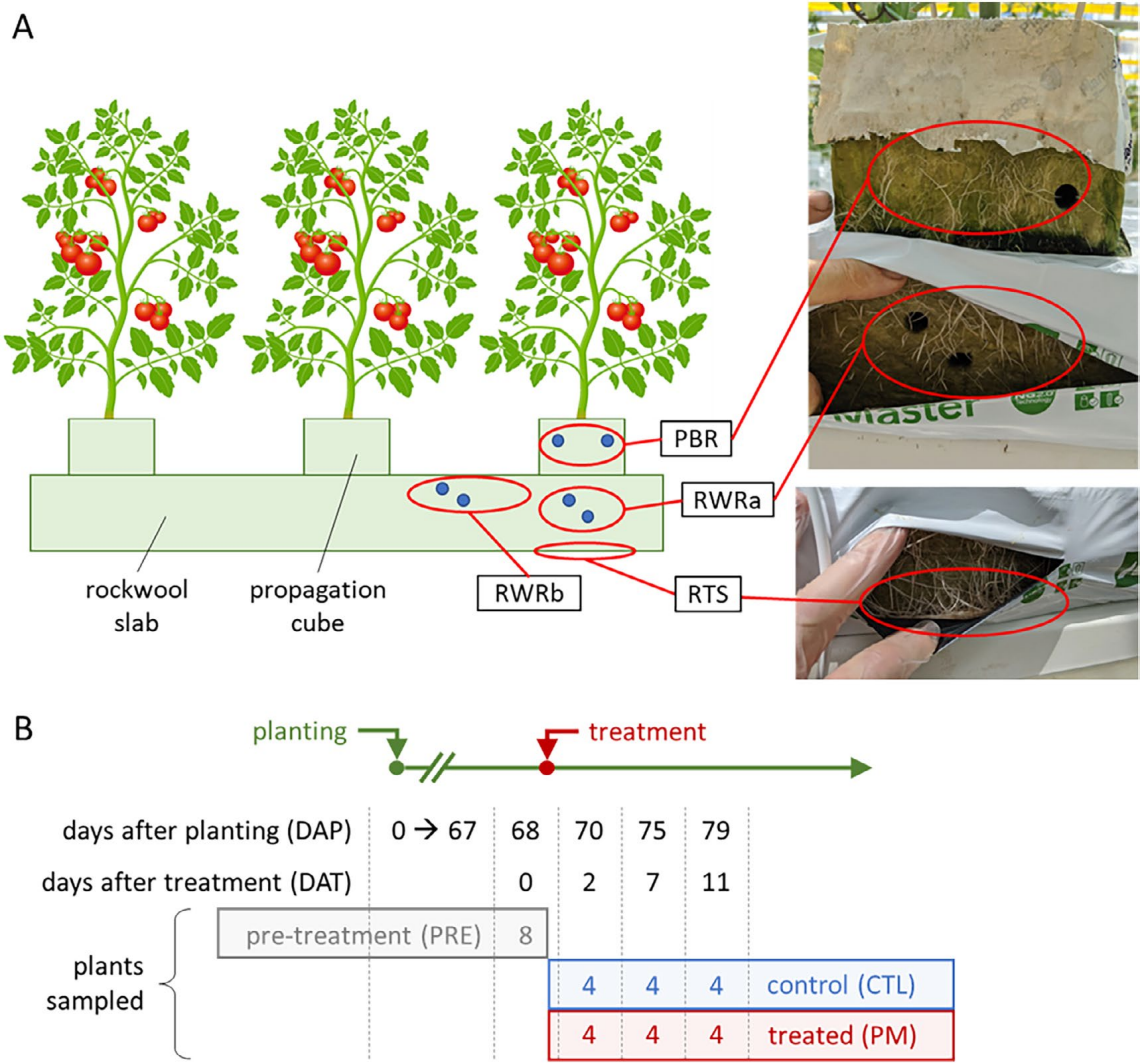
(A) Sampling positions in the hydroponic root zone. Horizontal cores were taken from the propagation cube (PBR), the rockwool slab below the stem of the plant (RWRa) and the upper part of the slab between plants (RWRb). Roots growing outside the slab were removed with forceps (RTS). (B) Schedule of treatment and sampling from pretreatment, control and treated plants. For each plant, samples from the four positions were pooled before DNA extraction.

The microbiome was sampled on four occasions between 68 and 79 DAP, when the plants were bearing mature trusses. Sampling was performed by extracting horizontal cores (diameter 9 mm, length 100 mm) from the rockwool slabs and propagation cubes, and by cutting bare roots growing outside the slab. Cores were taken from regular positions in the root zone (Figure 1A) which experience distinct environmental conditions likely to influence the composition of the microbial community (propagation cube; rockwool slab below plant stem; slab between plants; bare roots). Cores were stored in sterile 15 mL tubes which were chilled (~4°C) immediately after sampling and then frozen (−20°C) within 1 h until ready for processing.

Before processing, samples from four consistent positions in the root zone (PBR, RWRa, RWRb, RTS; Figure 1B) from the same plant were pooled, then added to sterile standard saline solution (NaCl 0.9% w/v, 100 mL) with a surfactant (Tween 80, 0.05% v/v) and placed on ice. Microorganisms were then isolated from the rockwool and root material by washing on an orbital shaker at 150 rpm for 90 min. Coarse material was then removed by passing through sterile 1.5 mm mesh, followed by centrifuging (500*g* for 2 min). Microorganisms were recovered from the wash suspension by centrifuging at 3000*g* for 30 min. This method enabled extraction of microbial cells from the rockwool matrix and root surfaces while minimising plant material. The recovery of viable cells was assessed by comparing plate counts of the original wash suspension and the final supernatant on nutrient agar (Oxoid, Thermo Fisher Scientific) and potato dextrose agar (Oxoid, Thermo Fisher Scientific). The pellet was resuspended, transferred to a 1.5 mL tube, centrifuged at 15,000*g* for 6 min and the supernatant discarded. The final pellet, containing cells and fine material produced by the wash process, was frozen and stored at −20°C until DNA extraction.

DNA was extracted from each sample pool using a DNeasy PowerSoil Pro kit (Qiagen, Australia) following the manufacturer's protocol, including initial incubation in solution C1 at 70°C for 10 min and elution in 100 μL sterile nuclease‐free water. To remove remaining contaminants, the extracted DNA was further purified by cold ethanol precipitation (0.1X vol 3 M sodium acetate, 3X vol 96% ethanol, incubated overnight at −80°C). Quantity and quality of the final DNA product were assessed with a NanoDrop spectrophotometer (Thermo Fisher Scientific, Australia), and dsDNA was quantified with a fluorometer (Quantus, Quantifluour dsDNA System, Promega, Australia). DNA was stored at −80°C until sequencing (Table S1).

### 2.3 Metabarcode Analysis

Microbial profiling of each sample was performed by Illumina MiSeq 2 × 250 bp paired‐end sequencing (Ramaciotti Centre for Genomics, Sydney, Australia) of the 16S rRNA gene V4 region (primers 515f–806r), and the 18S rRNA gene ITS1 region (primers ITS1F–ITS2), from which were obtained a total of 3,056,227 16S amplicon sequences (Table S2) and 1,367,139 ITS amplicon sequences (Table S4).

Sequences were processed with QIIME 2 2023.2 (Bolyen et al. 2019). Demultiplexed sequences were denoised with DADA2 (Callahan et al. 2016) via q2‐dada2 (Tables S2 and S4). Taxonomy was assigned to amplicon sequence variants (ASVs) using the q2‐feature‐classifier naïve Bayes taxonomy classifier (Bokulich et al. 2018) against the SILVA v138 rRNA gene database (Glöckner et al. 2017; Quast et al. 2013; Yilmaz et al. 2014) or UNITE version 9.0 (Kõljalg et al. 2020). 16S ASVs classified as either eukaryote, mitochondria or chloroplast were removed (Table S3). The 16S phylogenetic tree was created using the SATé‐enabled phylogenetic placement (SEPP) fragment insertion method (Janssen et al. 2018) and the Greengenes 13.8 reference phylogeny (McDonald et al. 2012). Metabarcode data were then analysed with the phyloseq package (McMurdie and Holmes 2013), ampvis2 (Andersen et al. 2018) and the tidyverse (Wickham et al. 2019). ASVs which may have originated from contamination during extraction were detected with the decontam package (Davis et al. 2018) using the frequency method. Fifteen possible contaminant 16S ASVs (1489 reads) and eight possible contaminant ITS ASVs (567 reads) were removed from further processing. An average of 553 unique 16S ASVs (SD 61.2; Table S3) and 71 ITS1 ASVs (SD 21.3; Table S5) were retained. 16S samples were rarefied to 54,697 reads and one sample below this number was dropped. ITS samples were rarefied to 22,544 reads and no samples were dropped.

Alpha diversity indices (Simpson, Shannon‐Wiener and Faith's phylogenetic diversity) were calculated with the vegan package (Oksanen et al. 2020) and means were compared using the Kruskall–Wallis rank sum test. Beta diversity was analysed by calculating unweighted or weighted Unifrac dissimilarity using the phyloseq package (McMurdie and Holmes 2013) and followed by adonis2 PERMANOVA (999 permutations) with Benjamini‐Hochberg adjustment of *p* values. Differences in relative abundance of taxonomic groups between treatments were assessed for significance using ANCOM‐BC2 (Lin and Peddada 2024).

## 3 Results

### 3.1 Bacterial Community Diversity and Resilience

**FIGURE 2:**
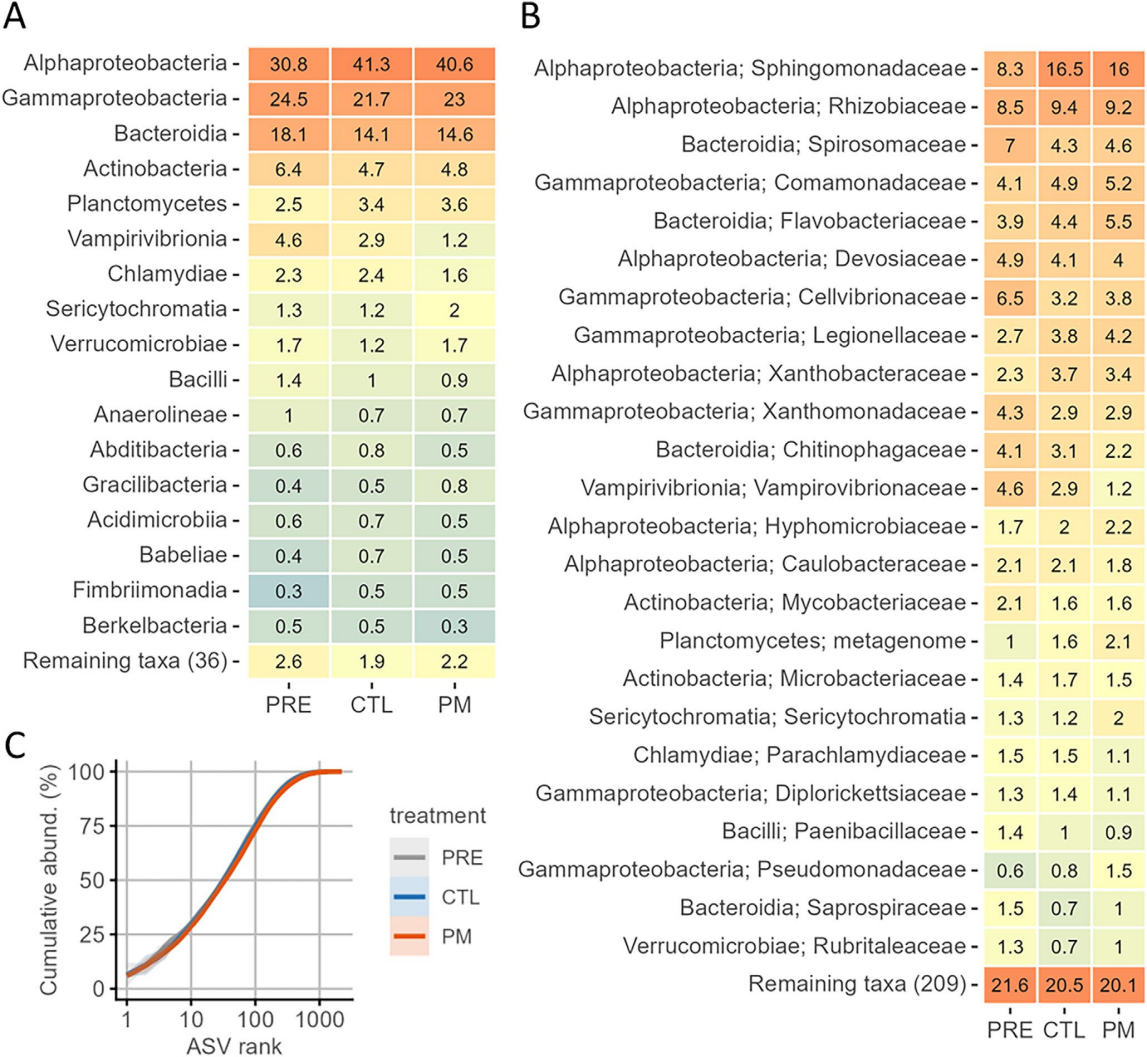
Relative abundance of bacteria in the hydroponic rockwool root zone (samples from four positions pooled) before treatment (PRE, *n* = 6), in untreated control plants (CTL, *n* = 12) and after treatment with a *Trichoderma*‐based biocontrol product (PM, *n* = 12). Heatmaps of the most abundant taxonomic groups at class level (A) and family level (B). Values are the mean relative abundance across all samples in each treatment group. (C) Rank abundance curve of ASVs by treatment group. (C) Rank abundance plot of ASV relative abundance in pretreated, control and treated microbiome samples.

The root zone of mature tomato plants was sampled at 68–79 DAP and relative abundance of bacteria was assessed from 16S rDNA amplicon sequences for pretreatment (PRE), treated (PM) and control (CTL) samples. The most abundant bacterial classes (Figure 2A) were Alphaproteobacteria (mean 38.9%, SD 8.4%), Gammaproteobacteria (mean 22.8%, SD 4.4%), Bacteroidia (mean 15.1%, SD 5.5%), Actinobacteria (mean 5.1%, SD 2.1%), Planctomycetes (mean 3.3%, SD 1.7%) and Vampirivibrionia (mean 2.6%, SD 3.0%; Cyanobacteria). The most abundant families (Figure 2B) were Sphingomondaceae (mean 14.6%, SD 6.2%), Rhizobiaceae (mean 9.1%, SD 2.0%), Spirosomaceae (mean 4.9%, SD 3.0%), Comamonadaceae (mean 4.9%, SD 1.4%) and Flavobacteriaceae (mean 4.8%, SD 2.7%). An average of 553 unique ASVs per sample (SD 61.2) were detected after quality filtering and removal of potential contaminants. Rank abundance comparison (Figure 2C) indicated that the 10 most dominant ASVs in the hydroponic system accounted for more than 25% of the total sequences. Together, these observations indicate that the bacterial microbiome was species‐rich but unevenly distributed.

ANCOM‐BC2 analysis did not identify any significant differences in relative abundance of taxa between treated and control plants (α = 0.05). There were, however, significant differences between early (pretreatment and 2 days after treatment) and later (7 and 10 days after treatment) samples (Table S6). ANCOM‐BC2 detected significant enrichment of *Novosphingobium* (log2‐fold‐change = 2.10, *q* < 0.001; Alphaproteobacteria) in later samples, while the most depleted groups with significant relative abundance (> 1%) were *Emticicia* (LFC = −1.72, *q* = 0.02; Bacteroidota) and *Cellvibrio* (LFC = −2.62, *q* = 0.0054; Gammaproteobacteria).

**FIGURE 3:**
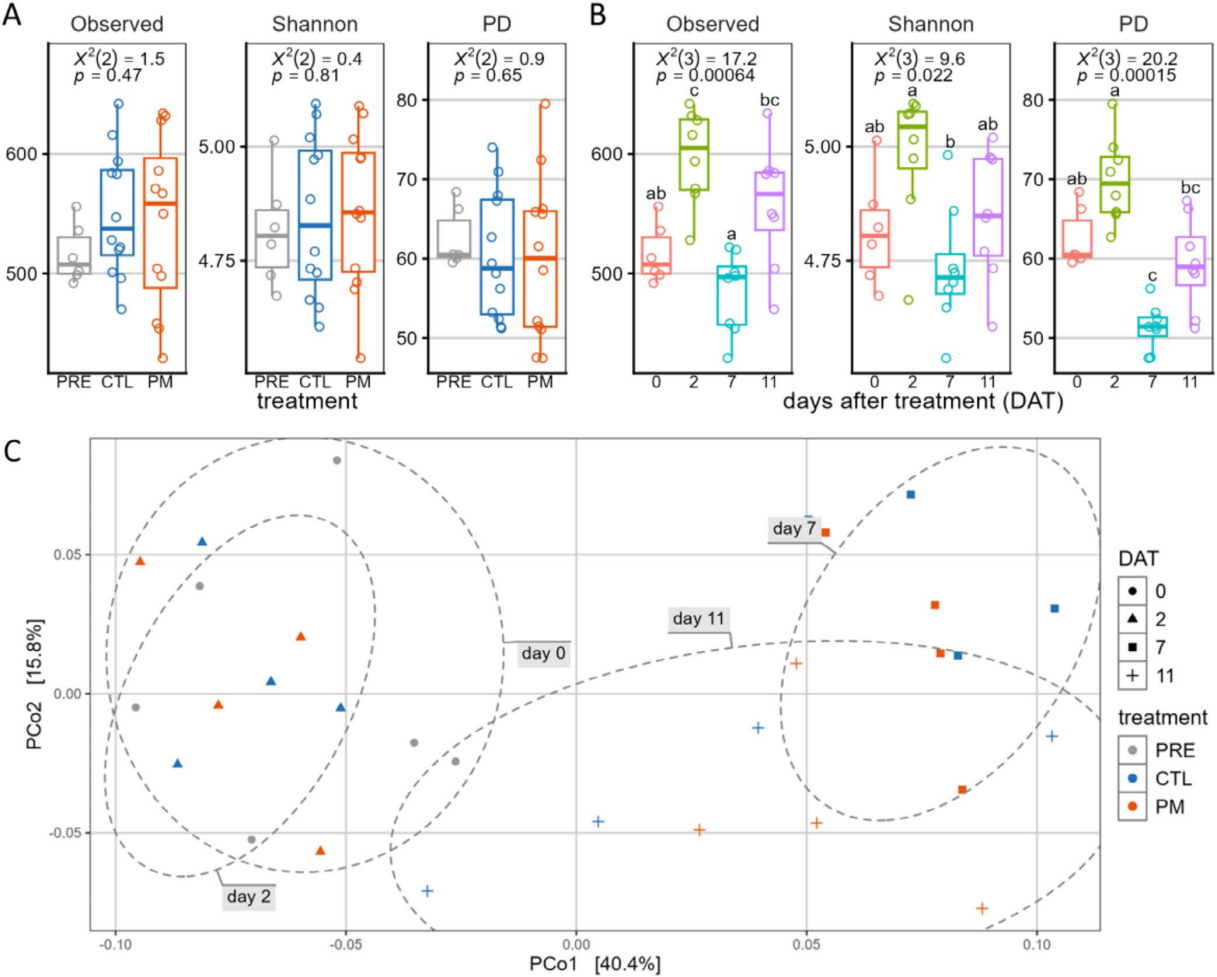
Comparison of bacterial alpha diversity by (A) treatment type (pretreatment, PRE, *n* = 6; untreated control, CTL, *n* = 12; *Trichoderma*‐treated, PM, *n* = 12) and by (B) days after treatment (*n* = 30). Alpha diversity indices were not significantly different when grouped by treatment group (A), but fluctuated over time when grouped by days after treatment (B). Results from Kruskal‐Wallis test are shown. Compact letter display denotes statistical similarity of groups (Dunn test, α = 0.05). (C) PCoA ordination of Unifrac distances with colours indicating treatment group and shapes indicating days after treatment. Ellipses group samples taken on the same day after treatment. There was no significant difference in microbiome composition between treated and untreated plants (PERMANOVA of weighted Unifrac distances, *n* = 24, *F* = 0.67, *p* = 0.66). Composition on Day 7 and Day 11 after treatment was significantly different to Day 0 and Day 1 (PERMANOVA of weighted Unifrac distances, *n* = 32, *F* = 5.81, *p* = 0.001).

Alpha diversity of the bacterial microbiome was not significantly different between *Trichoderma*‐treated and untreated plants (Figure 3A; for example, Shannon‐Wiener index, *Χ* ^2^ (2) = 0.3 *p* = 0.87). Similarly, there was no significant difference in microbiome composition between treated and untreated plants (Figure 3C; PERMANOVA of weighted Unifrac distances, *n* = 24, *F* = 0.67, *p* = 0.68). However, while no significant differences associated with treatment were detected, the diversity and composition of the microbiome did change over time. Alpha diversity fluctuated during the 12‐day sampling period (Figure 3B). In particular, there was a significant drop in diversity between Day 2 and Day 7 after treatment (e.g., Faith's phylogenetic diversity index, *Χ* ^2^ (3) = 20.1, *p* < 0.001) followed by return to diversity comparable to initial samples. Microbiome composition also changed significantly by Day 7 compared to earlier samples (e.g., PERMANOVA of weighted Unifrac, *n* = 32, *F* = 5.81, *p* = 0.001) and remained significantly different to initial samples.

### 3.2 Fungal Microbiome Diversity and Resilience to *Trichoderma* Biocontrol Treatment

**FIGURE 4:**
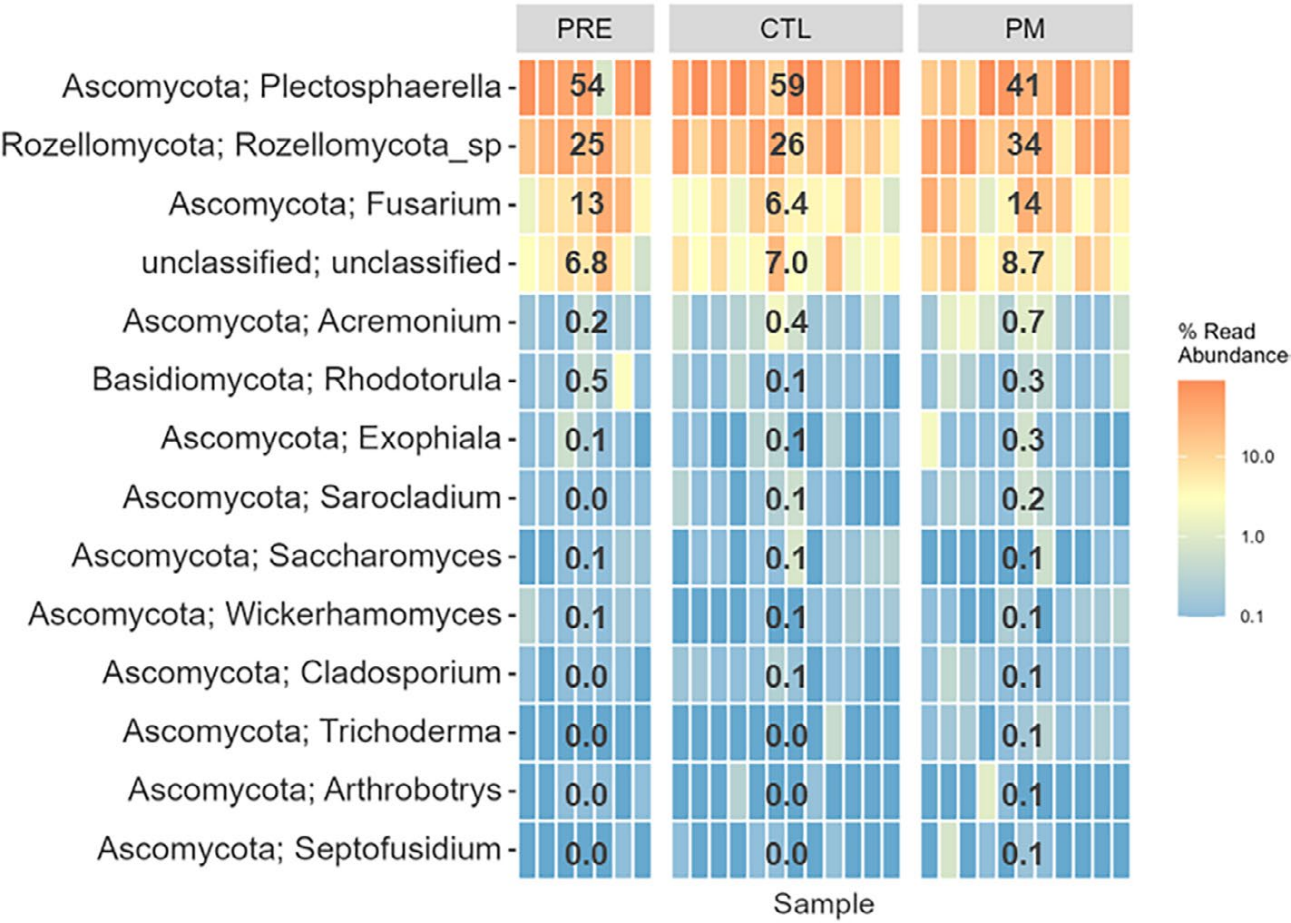
Heatmap of most abundant taxonomic groups at genus level grouped by treatment. Colours indicate relative abundance of reads per sample. Values are the mean relative abundance percentage within each treatment group.

Fungal microbiomes were characterised from ITS1 rDNA sequences. The fungal microbiome (Figure 4) was dominated by Ascomycota (overall mean relative abundance 64%, SD 22%) and Rozellomycota (mean 29%, SD 16%). Within the Ascomycota, *Plectosphaerella* (overall mean 51%, SD 27%) and *Fusarium* (mean 11%, SD 10%) were most common. A significant proportion of ITS1 ASVs were not classified (mean 7.6%, SD 7.1%). *Trichoderma* species were not detected before treatment. After treatment, *Trichoderma* was detected in all four treated slabs and in one control slab in low relative abundance (~0.1% of ITS1 reads). Only the genus *Trichoderma* was found to be significantly differentially abundant between treated and untreated plants (DESeq2, log_2_‐fold‐change = 5.0, *p‐adj* = 0.01). The apparent shift in relative abundance from *Plectosphaerella* to Rozellomycota in treated slabs (Figure 4) did not meet the threshold for significance. The discriminant power of the DESeq2 algorithm was probably limited by within‐group variation, which is evident for all abundant taxa in Figure 4.

**FIGURE 5:**
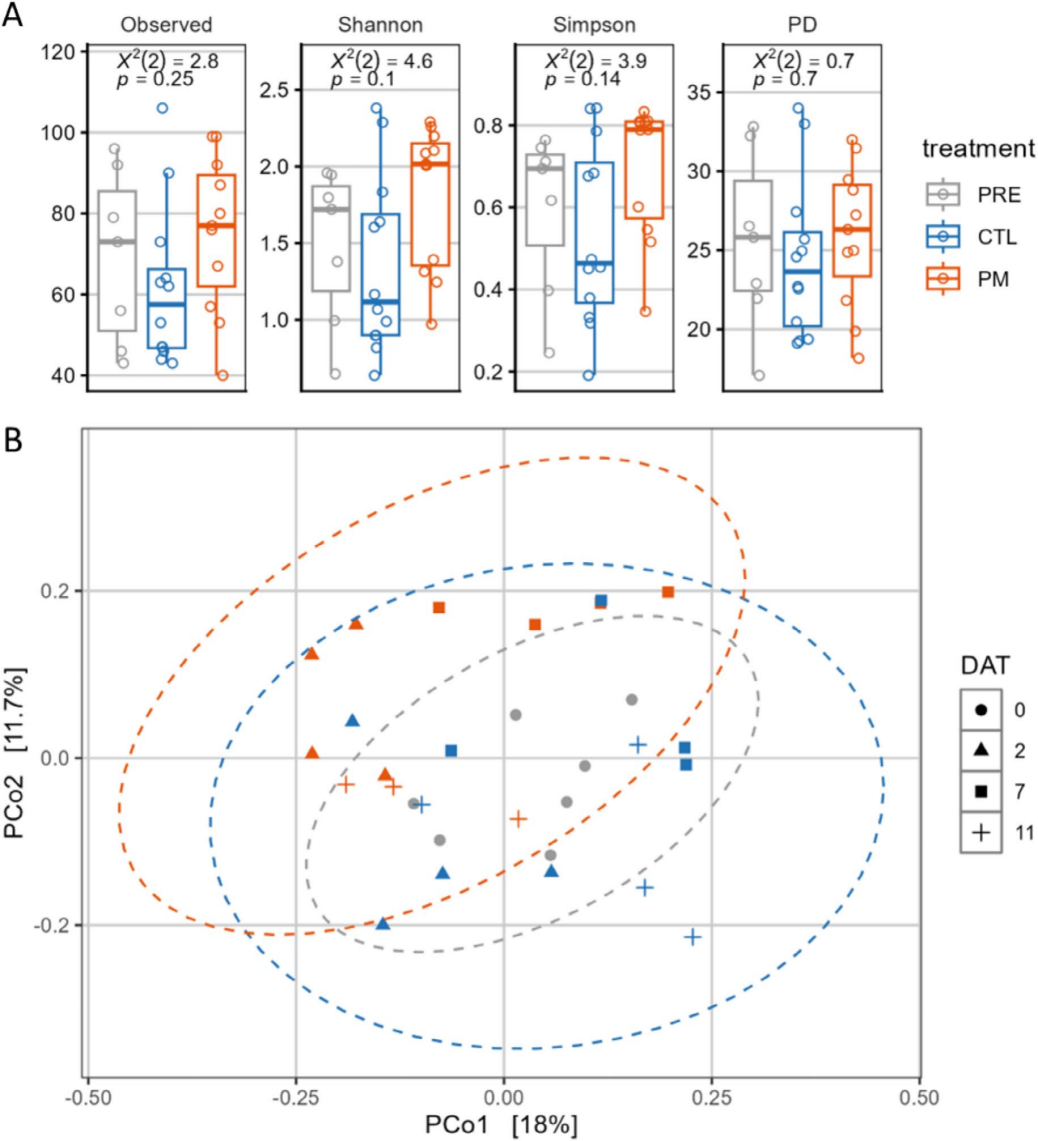
Diversity of fungal microbiomes in the root zone of tomato plants before treatment (PRE, *n* = 6), in untreated control plants (CTL, *n* = 12) and after treatment with a *Trichoderma*‐based biocontrol product (PM, *n* = 12). (A) Alpha diversity indices: Observed ASVs, Shannon‐Wiener index, Faith's phylogenetic diversity (PD) with results from Kruskal‐Wallis test. None of the diversity indices were significantly different among groups; however, pairwise comparison of Shannon indices between control and treated samples approached significance (*Χ* ^2^ (1) = 3.6, *p* = 0.056). (B) PCoA ordination of Unifrac distances with treatment group indicated by colour and treatment day indicated by shape. Unweighted phylogenetic composition of fungal microbiomes of treated plants was significantly different from pretreatment and control plants (PERMANOVA of Unifrac distances, *n* = 24, F = 2.31, *p* = 0.008). Ellipses indicate 95% confidence interval of a multivariate t‐distribution.

The median fungal alpha diversity was higher in *Trichoderma*‐treated slabs compared to controls (Figure 5A) but species richness (observed) and phylogenetic richness (Faith's phylogenetic diversity) were not significantly different (α = 0.05). However, pairwise comparison of mean Shannon‐Wiener diversity, which gives equal weight to evenness and richness, approached significance (CTL vs. PM, *Χ* ^2^ (1) = 3.6, *p* = 0.056). The unweighted composition of the microbiome (Figure 5B) was different in treated plants compared to untreated plants (PERMANOVA of Unifrac distances, *n* = 24, *F* = 2.31, *p* = 0.008); however, there was no difference between groups when taking relative abundance weighting into account (PERMANOVA of Weighted Unifrac distances, *n* = 24, *F* = 1.53, *p* = 0.195). This indicates that while there was a change in fungal species detected, such as the presence of the putative *Trichoderma* biocontrol species, this did not significantly affect the prevalence of the dominant species.

## 4 Discussion

Novel agricultural methods are emerging from our growing understanding of plant‐associated microbiomes, but the potential of microbial communities in hydroponic horticulture remains relatively unexplored. In protected cropping systems, attention is usually focused on detection and mitigation of potential plant pathogens or the putative benefits of plant growth‐promoting microorganisms (PGPMs). But hydroponic systems are colonised by a wide range of microorganisms, and the plants interact with microbes around them according to the genetic programming of millions of years of coevolution in soil. This study aimed to characterise the microbial ecology of the hydroponic root zone of mature tomato plants, including the response to inoculation with a popular biological control organism.

### 4.1 Bacterial Root Microbiome of Hydroponic Rockwool Tomatoes

In the rockwool root zone, we found a microbiome which was rich in bacterial species but strongly dominated by a few relatively abundant organisms, implying that most of the diversity consisted of low abundance taxa. This pattern of distribution is consistent with a homogeneous environment which provides a limited range of ecological niches, and may therefore be a distinguishing feature of the hydroponic microbiome, since it reflects the highly uniform glasshouse environment. Higher diversity, including rare taxa, provides functional redundancy which tends to enhance the stability of the community (Shade et al. 2012). While the number of species in the rockwool system was lower than in a typical soil, and was heavily weighted toward a small number of species, species richness does represent a reservoir of diverse organisms which may respond to changing conditions, such as changes in plant root activity or abiotic environment.

The most relatively abundant classes in the system, comprising over 80% of the microbiome, were Alphaprotobacteria, Gammaproteobacteria, Bacteroidota and Actinobacteria. Vargas et al. (2021) studied the root microbiome of tomatoes in a comparable rockwool system and found that, at a comparable crop age (9–17 weeks after planting), these taxa were also numerically dominant, comprising 55% of ASV reads. However, Bacilli were found to be the most dominant group, with overall relative abundance of 33%, which is a significant contrast to our results (mean relative abundance 1.1%, SD 0.5%). Anzalone et al. (2022) studied the root microbiome of tomatoes in an organic coconut fibre substrate and found that Proteobacteria, Bacteroidetes (Bacteroidota) and Actinobacteria represented over 80% of relative abundance, while Firmicutes (Bacillota) were only 2%. Anzalone et al. further compared the microbiome in coconut fibre to that of tomatoes grown in bagged agricultural soil in a greenhouse environment with drip irrigation and found that Proteobacteria, while abundant, were much less dominant in the soil rhizosphere, where the most relatively abundant phyla were Proteobacteria (30%), Bacteroidetes (30%), Firmicutes (~19%) and Actinobacteria (~8%). Studies of tomatoes in soil (Lee Díaz et al. 2024; Poudel et al. 2019) have found Proteobacteria and Actinobacteria to be the most relatively abundant phyla (~20%–30%), followed by Firmicutes (~12%). In comparison to hydroponic studies above and our observations this suggests that Actinobacteria and Firmicutes are commonly much more prevalent in soil than in soilless systems. However, a higher relative abundance of Firmicutes has been observed in hydroponic rockwool during the early stages of the crop when total microbial cell density is low (Vargas et al. 2021).

At family level, Sphingomonadaceae (primarily *Sphingobium*, *Novosphingobium* and *Sphingopyxis*) and Rhizobiaceae (primarily *Allorhizobium‐Neorhizobium‐Pararhizobium‐Rhizobium* species) were the most abundant groups in our experimental system. These taxa, along with *Streptomyces* and Microbacteriaceae, have been significantly associated with hairy root disease of tomatoes in hydroponic rockwool (Grunert et al. 2016; Vargas et al. 2021). The Vargas et al. (2021) study identified a high relative abundance of Paenibacillaceae (32%) in the rockwool microbiome, with smaller but significant populations of Rhizobiaceae (6%) and Flavobacteriaceae (4%). The most prominent families found by Grunert et al. (2016) in rockwool at a comparable crop age were Chitinophagaceae (7%), Xanthomonadaceae (7%), Microbacteriaceae (5%), Hyphomicrobiaceae (4%) and Flavobacteriaceae (2%). In the organic hydroponic root zone, Anzalone et al. (2022) found Flavobacteriaceae (~18%) and Pseudomonadaceae (10%) to be the most relatively abundant families, while Flavobacteriaceae (~15%) and Bacillaceae (~11%) were most numerous in bagged agricultural soil. Most of the most prominent families in our system (Sphingomonadaceae, Devosiaceae, Rhizobiaceae) or other studies above (Hyphomicrobiaceae, Paenibacillaceae, Xanthomonadaceae) include a large proportion of species capable of either gliding or flagellum motility (Podlesny et al. 2026) and have commonly been isolated from aquatic as well as soil and environments (Madin et al. 2020), suggesting traits likely to be beneficial in a hydroponic environment.

### 4.2 Fungal Microbiome of Hydroponic Rockwool Tomatoes

The diversity of fungal species in the rockwool root zone was significantly lower than that of bacteria, and the relative abundance of different taxa varied widely between samples. This is consistent with previous observations that the rockwool environment favours bacteria above fungi (Koohakan et al. 2004). *Plectosphaerella* (Ascomycota), Rozellomycota and Fusarium (Ascomycota) were the most abundant groups, together accounting for more than 90% of ITS1 reads. This is a strong contrast to hydroponic systems using organic substrates. Anzalone et al. (2022) identified more than 26 fungal families in the rhizosphere of tomatoes in hydroponic coconut fibre, including significant proportions of Trichocomaceae, Archaeortizomycetes and Cladosporiaceae. Interestingly, the Anzalone et al. (2022) study also found significantly higher fungal diversity in the rhizosphere of the plants in coconut fibre than plants in soil, despite similar diversity in the respective bulk media, suggesting that the organic substrate provides a highly favourable and diverse environment. Rozellomycota is a recently identified, basal phylum of fungi whose species have most often been reported in aquatic environments (Grossart et al. 2015). Rozellomycota and *Plectosphaerella* have been found to be enriched in the rhizosphere of tomatoes grown in soil with fertigation containing organic and mineral elements compared to irrigation with water alone (Marín‐Guirao et al. 2023). Both *Plectosphaerella* and *Fusarium* species have been found to be enriched in the rhizosphere of tomato plants grown in hydroponic coconut fibre compared to soil (Anzalone et al. 2022). *Plectosphaerella* is also abundant in the rhizosphere of hydroponically grown lettuce (Guevara et al. 2025) and can be pathogenic to plants (Usami and Katagiri 2017).

### 4.3 Effects of Treatment With *Trichoderma*‐Based Biocontrol Product

Due to their widely studied plant‐beneficial effects (Contreras‐Cornejo et al. 2016; Dutta et al. 2023), *Trichoderma* species have become attractive candidates for biological control of root pathogens and promotion of plant growth in commercial horticulture. Introducing PGPMs to the root zone can, however, affect the diversity of the resident community. Lee Díaz et al. (2024) applied two bacterial ( *Bacillus amyloliquefaciens* and *Pseudomonas azotoformans* ) and two fungal (*Trichoderma harzianum* and *Rhizophagus irregularis*) inoculants, both individually and combined as a synthetic community, to the roots of tomato seedlings in soil, and found that after 8 weeks of growth the bacterial root microbiome of plants treated with *T. harzianum* alone had significantly lower Shannon diversity and different composition to control plants. In our experimental system, a biocontrol product containing *Trichoderma* species was applied in solution to mature tomato plants in rockwool slabs. *Trichoderma* was not detected in the system before treatment, but was consistently detected in treated slabs until the end of the sampling period, albeit at very low levels. There was no significant difference in fungal diversity or composition between treated and untreated microbiomes, except for the presence of the *Trichoderma* species themselves. The capability to colonise an established microbiome and persist in the environment is an essential capability for effective biocontrol (Calvo‐Bado et al. 2006; Vallance et al. 2012). *Trichoderma* has been found to colonise the root surface and penetrate the epidermis in tomatoes (Harman et al. 2004) and cannabis (Scott and Punja 2023) and to persist for several months (Harman 2000). The low frequency of *Trichoderma* reads in this study may be explained by the sampling strategy, which focused on the rhizosphere, and did not aim to recover endophytic organisms.

No significant differences in alpha diversity or composition of the bacterial community were associated with the *Trichoderma* treatment. Previous studies of hydroponic systems have observed a similar resilience during colonisation by a foreign organism. Vallance et al. (2012) found that an oomycete biocontrol agent, *Pythium oligandrum*, had only a transient effect on the structure of bacterial communities, and suggested that this was mainly due to the stability of the microbiome. Calvo‐Bado et al. (2006) found that infection by a pathogenic *Pythium* species had only a minor influence on incumbent bacterial and fungal communities in the root zone of tomatoes in rockwool.

### 4.4 Temporal Variation in Bacterial Diversity

While there was no detected contrast in diversity associated with treatment, the bacterial microbiome did change distinctly during the 11‐day sampling period. Alpha diversity fluctuated and community composition was significantly different in later samples. Similar temporal dynamics have been observed in other studies; however, these studies sampled at intervals of a month or longer. Vallance et al. (2012) investigated the effect of the biocontrol oomycete Pythium oligandrum on a hydroponic tomato rhizosphere and observed a temporary change in bacterial community composition associated with treatment, along with a persistent, progressive change between monthly samples over the 6‐month life of the crop. This study did not report alpha diversity. Lin et al. (2021) found a strong correlation between composition of the root microbiome and plant developmental stage in a melon crop in a hydroponic system. The Vargas et al. (2021) study of tomatoes in hydroponic rockwool found that Shannon diversity increased over the life of the crop (samples taken in February, May, July, August and November), and that crop stage explained most variation in community composition. Subsequent analysis of the 16S data from this study identified groups of bacteria whose early relative abundance was associated with the incidence of hairy root disease in later stages of growth (Huo et al. 2025).

Variation in plant activity has a strong effect on the microbial environment via the composition and quantity of root exudates (Alsanius et al. 2019; Zhalnina et al. 2018). Beneduce et al. (2024) assessed the effect of different plant nutritional regimes on the microbiome of tomatoes in hydroponic rockwool and found that the plant development stage had a stronger influence than nutrition on community composition and relative abundance of nitrogen cycling genes. Direct comparison with the above studies is not feasible due to differences in sampling intervals and the range of different metrics used to assess diversity, so we are unable to conclude whether the fluctuation in alpha diversity and change in beta diversity we observed are a common feature of the hydroponic environment or are specific to our experimental system.

In our system, Sphingomonadaceae were significantly enriched in later samples, with the genus *Novosphingobium* increasing most prominently in abundance. Sphingomonadaceae are known for their capability to catabolise complex organic compounds, such as aromatic hydrocarbons, which derive from an array of plasmid‐based genes (D'Argenio et al. 2014). In contrast, *Cellvibrio* and *Emticicia* were the most prevalent genera which were significantly depleted in later samples. Species in these genera are known for an ability to degrade complex polysaccharides including cellulose and chitin. These distinct fluctuations in abundance of groups with contrasting metabolic capability suggest that variation in organic material in the root zone is linked to changes in the composition of the microbiome over time; however, the scope of this study was not sufficient to verify this relationship.

### 4.5 Practical Implications for Hydroponic Systems

Plant‐beneficial features of the hydroponic microbiome, including a surprising diversity and community structure which is resilient to disturbance, are gradually becoming apparent from ongoing investigations. A resilient and functionally diverse root microbiome can prevent establishment of pathogens by competitive exclusion (Bais et al. 2006; Shade et al. 2012; Wei et al. 2015). The distribution of species in the rockwool system was, however, highly uneven, which can reduce microbiome stability (Wittebolle et al. 2009). Nurturing the abundance of a wider range of plant‐associated bacteria which are already present is therefore a potential strategy to mitigate the risk of root‐borne pathogens in rockwool. Hydroponic systems provide a unique opportunity to manipulate the root microbiome, because they enable much more direct control over the environment in the root zone than in soil crops. For example, selection of an organic growth substrate, such as coconut fibre, significantly increases the higher abundance and diversity of fungi compared to mineral substrates like rockwool (Anzalone et al. 2022). The fertigation solution, which is predominantly or entirely composed of mineral ions, can be enriched with more diverse organic compounds to increase the abundance of a wider variety of plant‐associated microorganisms. Greater understanding of the organisms that flourish in this environment can guide management practices to control and monitor the microbiome.

The prevalence and relative abundance of species found in hydroponic systems is distinctly different to the rhizosphere of similar crops in soil systems. Prominent taxa identified in hydroponic systems are usually found in association with plants, but also share traits suited to survival in aquatic environments. Growers are seeking biological alternatives to chemical treatments for control of plant disease and growth promotion. However, protected cropping is presently only a small (albeit growing) subsector of agriculture, so there has been less economic incentive for suppliers to develop, test and register hydroponic‐specific products (Alsanius and Wohanka 2019). Considering the differences between the environments, there is a risk that a biological treatment tested with soil crops is not well suited for soilless systems or may have adverse effects. If a putative beneficial organism cannot successfully compete with the incumbent community and persist in the environment, or requires frequent reapplication, its beneficial effects are likely to be negligible (Vallance et al. 2012). The likely efficacy of a biological product can be evaluated prior to field testing by considering whether the constituent organism(s) are adapted to an aquatic environment based on their ecological traits. Moreover, broader analysis of the predominant taxa in hydroponic systems can help to identify beneficial organisms which are specifically suited to the hydroponic environment rather than soil.

While the present study did not address plant factors, crop performance remains the primary concern of growers. Identifying links between plant health and the microbiome is therefore an important target for investigation. To quantify the effect of root‐colonising biocontrol agents such as *Trichoderma*, a more specific assay method should be used. Rootstock and scion varieties influence plant‐microbiome interactions, so a broader scope of study is needed to develop a general understanding of commercial hydroponic systems. This study was limited to a single plant variety and a short sampling period of 11 days. Since microbiome composition has been observed to change throughout the life of a hydroponic rockwool crop (Vargas et al. 2021), a longer sampling period than this preliminary study is necessary for greater understanding of the ecology of the system. Pooling samples to obtain suitable quality of DNA for sequencing reduced the statistical power of the results.

## Author Contributions

**Phil Thomas:** conceptualization, methodology, data curation, investigation, formal analysis, visualization, project administration, writing – original draft, writing – review and editing. **Brian Sindel:** funding acquisition, supervision, writing – review and editing. **Gal Winter:** conceptualization, funding acquisition, supervision, project administration, resources, writing – review and editing.

## Funding

This work was supported by the Australian Government.

## Conflicts of Interest

The authors declare no conflicts of interest.

## Supporting information


**Table S1:** Quality and quantity of DNA extracted from rockwool samples. Treatments: pretreatment (PRE), control (CTL), PlantMate (PM). Sample day (days after planting, DAP). DNA quality measures based on ratios of absorbance at 260, 280 and 230 nm. DNA concentration measured by fluorometer.
**Table S2:** Statistics for DADA2 denoising and quality filtering of rRNA gene 16S V4 sequences from rockwool samples. MiSeq reads: number of FASTQ sequences. Quality reads: number of reads passing DADA2 quality filter (using default threshold of < 2 estimated base errors). Quality reads as percentage of input reads. Denoised reads: after application of the DADA2 error model. Nonchimeric reads: after chimera detection and removal. Final reads, after filtering, denoising and chimera removal, as a percentage of input reads.
**Table S3:** Statistics for filtering during qiime processing for rockwool 16S samples. Filtered reads: after removal of ASVs classified as eukaryote, mitochondria or chloroplast. The lowest depth sample (C02p05a) was dropped from further processing.
**Table S4:** Statistics for DADA2 denoising and quality filtering of 18S rRNA gene ITS1 sequences from rockwool samples. MiSeq reads: number of FASTQ sequences. Quality reads: number of reads passing DADA2 quality filter (using default threshold of < 2 estimated base errors). Quality reads as percentage of input reads. Denoised reads: after application of the DADA2 error model. Nonchimeric reads: after chimera detection and removal. Final reads, after filtering, denoising and chimera removal, as a percentage of input reads.
**Table S5:** Statistics for filtering during qiime processing for rockwool ITS samples. Filtered reads: after removal of ASVs classified as mitochondria or chloroplast.
**Table S6:** Differential abundance at genus level for taxa with > 1% overall relative abundance. Log‐fold‐change (LFC), LFC standard error (LFC SE) and q‐value were calculated by ANCOM‐BC2 with default parameters comparing relative abundance before treatment (Day 0) to the given day after treatment (DAP).

## Data Availability

The data that support the findings of this study are openly available in European Nucleotide Archive (ENA) at https://www.ebi.ac.uk/ena/browser/view/PRJEB90498, reference number PRJEB90498.
